# Increased mortality in patients with secondary diagnosis of atrial fibrillation: Report from Chinese AF registry

**DOI:** 10.1111/anec.12774

**Published:** 2020-07-15

**Authors:** Xing‐Hui Shao, Yan‐Min Yang, Jun Zhu, Li‐Tian Yu, Li‐Sheng Liu

**Affiliations:** ^1^ Emergency and Intensive Care Center Fuwai Hospital Chinese Academy Of Medical Sciences Beijing China

**Keywords:** atrial fibrillation, diagnosis, emergency medicine, heart failure, mortality

## Abstract

**Background:**

The relationship between mortality and the primary diagnosis in AF patients is poorly recognized. The purpose of the study is to compare the differences on mortality in patients with a primary or secondary diagnosis of AF and to identify risk factors amenable to treatment.

**Methods:**

This was a prospective cohort study using data from the Chinese AF registry. For admitted patients, a follow‐up was completed to obtain the outcomes during 1 year.

**Results:**

A total of 2015 patients with confirmed AF were included. AF was the primary diagnosis in 40.9% (*n* = 825) of them. 78.9% (*n* = 939) of the secondary AF diagnosis patients and 55.5% (*n* = 458) of the primary AF diagnosis patients were sustained AF. Compared with primary AF diagnosis group, the secondary AF diagnosis group was older with more comorbidities. At 1 year, the unadjusted mortality was much higher in the secondary AF diagnosis groups compared with the primary AF diagnosis groups. In Cox regression analysis with adjustment for confounding factors, patients with secondary AF diagnosis were associated with an increased mortality (relative risk 1.723; 95% CI: 1.283 to 2.315, *p* < .001). On multivariate analysis, age ≥ 75, LVSD, COPD, and diabetes were independent predictors of mortality in patients with primary AF diagnosis, while for the secondary AF diagnosis group, the risk factors were age ≥ 75, heart failure, and previous history of stroke.

**Conclusions:**

Patients presenting to ED with secondary diagnosis of AF were suffering from higher mortality risks compared with primary AF diagnosis patients. Physicians should distinguish these two groups in clinical practice.

## 1 INTRODUCTION

Atrial fibrillation (AF) is the most common sustained cardiac rhythm disorder associated with increased morbidity and mortality (Lip, Brechin, & Lane, 2012). It is recognized as a considerable healthcare burden, because of an aging population and the strong relevancy between AF and its concomitant diseases such as stroke, myocardial infarction, and congestive heart failure (Airaksinen et al., 2013; Camm et al., 2010; Dickinson, Chen, & Francis, 2014). Data from the Framingham study demonstrated a 1.5‐fold to 1.9‐fold risk of mortality in patients with AF after adjustment for the preexisting cardiovascular conditions (Benjamin et al., 1998). Subsequently, numerous studies have drawn the same conclusion in various populations (Badheka et al., 2011; Jabre et al., 2011). However, there is relatively little research concerning on the relationship between mortality and the primary diagnosis of these patients. Moreover, the clinical profile, therapeutic management, and outcomes of AF patients with a different primary diagnosis were poorly recognized. Therefore, we performed a prospective trial to compare the difference on mortality between primary AF diagnosis patients and other primary diagnosis patients in a group of confirmed AF patients.

## 2 MATERIALS AND METHODS

The Chinese AF registry was a multicenter, prospective, observational study enrolled patients from 20 participating hospitals between November 2008 and October 2011. Patients who presented to an emergency department (ED) with atrial fibrillation or flutter were recruited in the study. Based on their primary diagnosis, admitted patients were divided into two groups: AF/flutter or the other. For all the individuals, one‐year follow‐up was performed. The study was approved by the ethics committees of each institution, and informed consent was obtained from all individual participants included in the study.

All participating centers were encouraged to enroll consecutive patients to minimize selection bias. The inclusion criteria were as follows: identification of patients using electronic hospital databases recording emergency department diagnoses, review of electrocardiograms, and telemetry recordings from the emergency department and direct screening by emergency department staff. Each hospital admission is assigned one primary diagnosis and up to nine secondary diagnoses when discharged. The primary diagnosis describes the main cause of hospitalization. Baseline data collected included patient demographics, visit characteristics, medical history, medication and interventions in ED, and hospital discharge information. Follow‐up was carried out at one year from time of enrollment by telephone interview. All‐cause mortality and the primary reason for death were identified. We defined heart failure, stroke and pulmonary embolism, and myocardial infarction as cardiovascular mortality. All treatment decisions were left to the discretion of the treating physician.

For blood pressure and heart rate, initial data at registration were documented. Body mass index (BMI) (weight [KG]/height[m]^2^) was calculated using the weight and height measured at enrollment. The definitions of AF types were in accordance with American College of Cardiology/American Heart Association/ European Society of Cardiology 2006 guidelines for AF (Fuster et al., 2006). We combined persistent AF and permanent AF as sustain AF. The risk stratification scheme CHADS2 based on a scoring systemic in which 2 points are assigned for a history of stroke and transient ischemic attack and 1 point each is assigned for age more than 75 year, a history of hypertension, diabetes, or recent clinical heart failure or impaired left ventricular systolic function.

Data were collected on a standardized case report form (CRF) through searching medical record and patient interview from each center. The CRF was sent to Fuwai hospital by fax at the earliest opportunity. Using a validation plan, integrated in the data entry software, data were checked for missing or contradictory entries and values out of the normal range. Additional edit checks were performed by the staff in Fuwai hospital.

### 2.1 Statistical analysis

Categorical variables were expressed as frequencies and percentage, and the normally distributed continuous variables were presented as mean with standard deviation (*SD*). Different patient strata were compared by chi‐squared tests for categorical variables and by the *t* test for continuous variables. Cox proportional hazards regression analyses were used to identify whether patients with AF as the secondary diagnosis were associated with increased 1‐year mortality and the independent predictors of mortality in each group. The models included age (as a second‐degree polynomial), sex, body mass index (as a second‐degree polynomial), type of AF, history of myocardial infarction, coronary artery disease, heart failure, hypertension, diabetes, previous stroke/TIA, history of left ventricular systolic dysfunction, left ventricular hypertrophy, chronic obstructive pulmonary disease, valvular heart diseases, prior major bleeding, sleep apnea, hyperthyroidism, smoke and medications (including ACE inhibitors or angiotensin II receptor blockers (ARB), β‐blockers, calcium channel blockers (CCB), digoxin, diuretics, anticoagulants, aspirin, or platelet inhibitors and lipid‐lowering drugs). Kaplan–Meier curves were constructed for time to event and were compared by log‐rank test. Stratification was performed by the type of AF and whether AF was the primary diagnosis at admission in order to meet model assumptions. The data were analyzed with SPSS 17.0, and a 2‐sided *p* value < .05 was considered statistically significant.

## 3 RESULTS

The Chinese AF registry has recruited 2016 patients with confirmed atrial fibrillation or atrial flutter. After excluded 1 patient with incomplete baseline data, a total of 2015 patients with a mean age of 68.46 ± 13.28 years were enrolled in the final analysis: of 1,190 patients in the secondary AF diagnosis group, 939 (78.9%) were sustain AF (group 1), 251 were non‐sustain AF (group 2), and of 825 patients in the primary AF diagnosis group, 458 (55.5%) were sustain AF (group 3), 367 were non‐sustain AF (group 4).

Patient demographics, past medical history, and medication during the ED visit of the four groups are demonstrated in Table 1. Compared with the two primary AF diagnosis groups, secondary AF diagnosis groups, on average, were much older, and more likely to have a history of myocardial infarction, coronary artery disease, heart failure, left ventricular systolic dysfunction (LVSD), previous stroke/TIA or chronic obstructive pulmonary disease (COPD), and had a similar prevalence of hypertension, diabetes, hyperthyroidism, or smoking.

**TABLE 1:** Overview of data from patients with sustain or non‐sustain AF and an alternative primary ED diagnosis

Demography	Secondary AF diagnosis (*n* = 1,190)		Primary AF diagnosis (*n* = 825)		*p* value
	Sustain AF (*n* = 939) Group 1	Non‐sustain AF (*n* = 251) Group 2	Sustain AF (*n* = 458) Group 3	Non‐sustain AF (*n* = 367) Group 4	
Age (years)	69.83 (13.15)	69.48 13.12)	67.58 (12.92)	65.35 (13.63)	<.001
Female gender	518 (55.2)	138 (55.0)	255 (55.7)	193 (52.6)	.820
BMI (kg/m2)	23.15 (3.73)	23.44 (3.30)	23.66 (3.47)	24.38 (3.49)	<.001
Systolic BP (mmHg)	133.77 (24.03)	134.49 (25.92)	130.90 (22.11)	126.59 (20.05)	<.001
Diastolic BP (mmHg)	80.74 (15.96)	80.31 (15.99)	79.58 (13.25)	77.94 (12.04)	.019
Heart rate (time/minute)	97.12 (26.67)	98.59 (28.70)	105.55 (31.92)	110.81 (30.37)	<.001
Mean CHADS_2_ score^a^	2.16 (1.40)	1.85 (1.36)	1.59 (1.31)	1.33 (1.21)	<.001
Medical history					
Myocardial infarction	86 (9.2)	28 (11.2)	18 (3.9)	16 (4.4)	<.001
Coronary artery disease	432 (46.0)	122 (48.6)	178 (38.9)	111 (30.2)	<.001
Heart failure	512 (54.5)	72 (28.7)	127 (27.7)	42 (11.4)	<.001
Hypertension	513 (54.6)	143 (57.0)	249 (54.4)	213 (58.0)	.637
LVH	196 (20.9)	33 (13.2)	63 (13.8)	37 (10.1)	<.001
Stroke/TIA	221 (23.5)	47 (18.7)	69 (15.1)	42 (11.4)	<.001
Sleep apnea	35 (3.7)	9 (3.6)	11 (2.4)	15 (4.1)	.538
Smoke	219 (23.3)	45 (17.9)	86 (18.8)	83 (22.6)	.110
LVSD	259 (27.6)	37 (14.7)	66 (14.4)	23 (6.3)	<.001
Cognitive disorder	30 (3.2)	6 (2.4)	4 (0.9)	4 (1.1)	.016
COPD	144 (15.4)	32 (12.7)	38 (8.3)	22 (6.0)	<.001
Diabetes	162 (17.3)	43 (17.1)	59 (12.0)	48 (13.1)	.079
Hyperthyroidism	24 (2.6)	8 (3.2)	23 (5.0)	11 (3.0)	.110
Valvular heart disease	215 (22.9)	18 (7.2)	81 (17.7)	22 (6.0)	<.001
Major bleeding	31 (3.3)	5 (2.0)	7 (1.5)	5 (2.4)	.083
Medication					
ACE inhibitor	282 (30)	59 (23.5)	124 (27.1)	68 (18.5)	<.001
ARB	169 (18.0)	50 (19.9)	76 (16.6)	75 (20.4)	.476
β‐blocker	452 (48.1)	110 (43.8)	257 (56.1)	196 (53.4)	.004
CCB	237 (25.2)	82 (32.7)	130 (28.4)	124 (33.8)	.007
Diuretics	522 (55.6)	80 (31.9)	188 (41.0)	67 (18.3)	<.001
Digoxin	451 (48.0)	53 (21.1)	166 (36.2)	48 (13.1)	<.001
Lipid‐lowering medication	242 (25.8)	76 (30.3)	119 (26)	91 (24.8)	.452
Aspirin or platelet inhibitor	592 (63)	160 (63.7)	296 (64.6)	233 (63.5)	.953
Oral anticoagulant	196 (20.9)	42 (16.7)	83 (18.1)	54 (14.7)	.057
Outcomes					
1‐year all‐cause mortality	179 (19.3)	34 (13.9)	46 (10.1)	18 (5.0)	<.001
Cardiovascular mortality	98 (54.7)	18 (52.9)	29 (63.0)	7 (38.9)	.245

Note

Data are number (%) or mean (*SD*).

Abbreviations: ARB, angiotensin II receptor blockers; BP, blood pressure; CCB, calcium channel blocker; COPD, chronic obstructive pulmonary disease; LVH, left ventricular hypertrophy; LVSD, left ventricular systolic dysfunction; TIA, transient ischemic attack.

^a^ A risk stratification scheme for atrial fibrillation. A score of 0–6 is derived based on the following factors: congestive heart failure (1 point); hypertension (1 point); age＞75 years (1 point); diabetes mellitus (1 point); and previous stroke or TIA (2 point).

**FIGURE 1:**
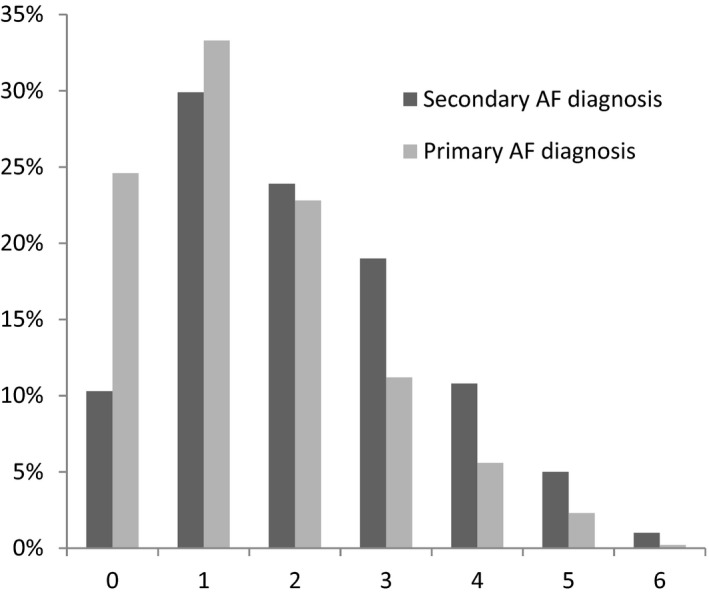
The proportion of population by CHADS_2_ score

At enrollment, blood pressure was higher, but heart rate was much lower in the two secondary AF diagnosis groups compared with the primary AF diagnosis groups. The mean CHADS_2_ score was higher in the secondary AF diagnosis group than primary AF diagnosis group. As the increasing score of CHADS_2_ marking scheme, the proportion of patients with a secondary diagnosis of AF was increasing (Figure 1). In patients with secondary diagnosis of AF, the top 7 definite primary ED diagnosis are listed in Table 2, along with the 7 most common presenting chief complaints.

**TABLE 2:** Presenting Primary ED Diagnoses and Chief Complaint of 1,190 Patients with Atrial Fibrillation and an Alternative Primary ED Diagnosis

Characteristic	*N*	(%)	95% CI
Definite primary ED diagnosis (*n* = 756)			
Heart failure	366	48.4	44.8–52.0
Stroke	80	10.6	8.4–12.8
Pneumonia	44	5.8	4.1–7.5
Infection	39	5.2	3.6–6.8
Acute coronary syndrome	34	4.5	3.0–6.0
Hypertension	28	3.7	2.4–5.0
Coronary heart disease	27	3.6	2.3–4.9
Chief complaint of indefinite ED diagnosis (*n* = 434)			
Palpitation	89	20.5	16.7–24.3
Fever	83	19.1	15.4–22.8
Dyspnea	80	18.4	14.8–22.0
Dizzy	68	15.7	12.3–19.1
Chest distress	45	10.4	7.5–13.3
Abdominal pain/Chest pain	22	5.1	3.0–7.2
Weakness/Fatigue	10	2.3	0.9–3.7

During hospitalization, ACE inhibitors, diuretics, digoxin were all given significantly more often in patients with secondary diagnosis of AF, whereas they less frequently received β‐blocker and CCB, especially in group 1. There was no difference between the groups with regard to ARB, lipid‐lowering medication, or antithrombotic therapy (Table 1).

**FIGURE 2:**
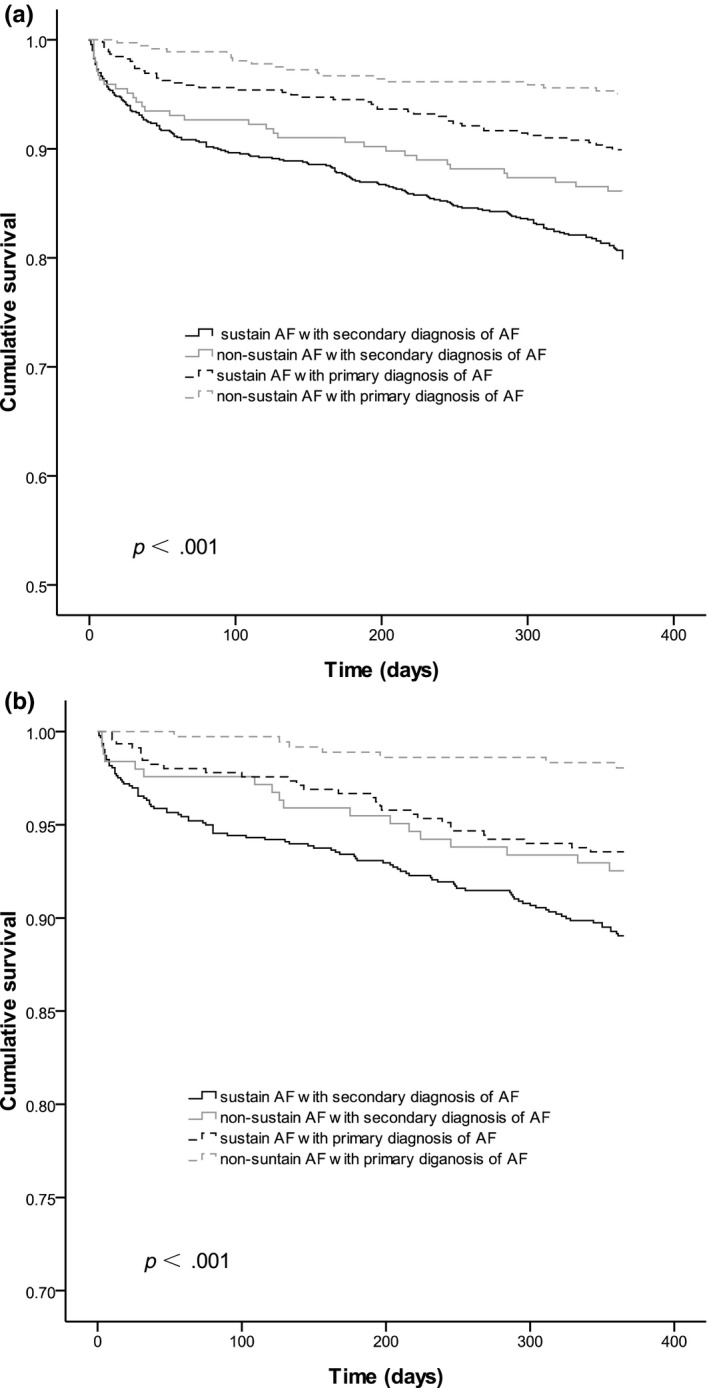
Kaplan–Meier estimates of cumulative survival of endpoint in each group (a: all‐cause mortality, b: cardiovascular mortality)

**FIGURE 3:**
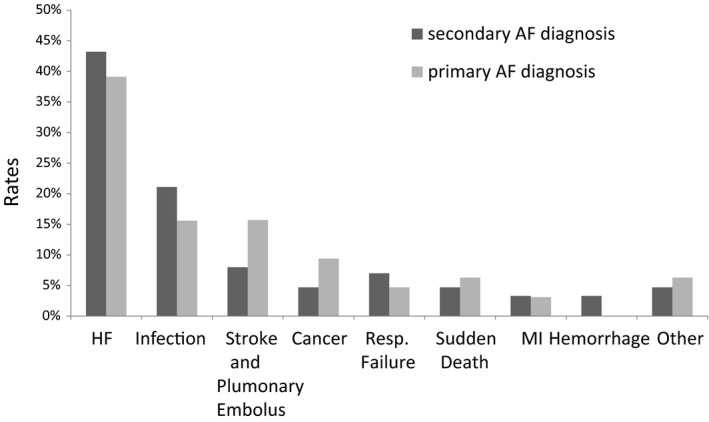
Cause of death in patients divided into 2 groups of primary diagnosis

The crude results indicated that all‐cause mortality was significantly higher in secondary AF diagnosis group than in the primary AF diagnosis group at 1 year, while the cardiovascular mortality was no significant difference between these groups (Table 1). The unadjusted absolute risk augment of death within 1 year was 14.3% in group 1 compared with group 4; Kaplan–Meier cumulative hazard curves are shown in Figure 2. After adjustment for the confounders, 1‐year mortality was still significantly increased in secondary AF diagnosis group, with a relative risk of 1.72 (95% CI 1.28–2.32; *p* < .001) compared with primary AF diagnosis group. The all‐cause mortality risk showed no heterogeneity for a large number of subgroups analyzed, except for patients with a history of COPD, among whom there was a tendency toward decreased risk in secondary AF diagnosis group. In regard to the cardiovascular mortality, patients with a history of COPD and without a history of HF were associated with decreased risk in secondary AF diagnosis group (Table 3). Heart failure, infection or stroke and pulmonary embolus were the most common causes of death among both of the two groups (Figure 3).

**TABLE 3:** Adjusted relative risk of 1‐year mortality in patients on secondary AF diagnosis versus primary AF diagnosis

	Proportio*n* (%)	All‐cause mortality		Cardiovascular mortality	
		Relative risk	95% CI	Relative risk	95% CI
All	100%	1.723	1.283–2.315	1.384	0.927–2.065
Male	45.2	2.308	1.463–3.641	2.331	1.111–4.889
Female	54.8	1.367	0.924–2.022	0.988	0.606–1.609
<75	66.0	1.740	1.070–2.829	1.448	0.794–2.639
≥75	34.0	1.611	1.112–2.336	1.286	0.754–2.193
Non‐sustain AF	30.7	1.824	0.982–3.386	2.277	0.868–5.976
Sustain AF	69.3	1.669	1.196–2.328	1.243	0.804–1.921
No History of MI	92.7	1.626	1.203–2.198	1.222	0.811–1.844
history of MI	7.3	6.747	0.816–55.807	^a^	
No History of CAD	58.2	1.735	1.144–2.631	1.456	0.842–2.516
history of CAD	41.8	1.544	1.013–2.354	1.126	0.621–2.042
No History of HF	62.6	1.483	1.025–2.146	0.943	0.528–1.685
history of HF	37.4	2.302	1.349–3.929	2.027	1.092–3.762
NO history of HTN	44.5	1.847	1.125–3.031	1.254	0.654–2.403
History of HTN	55.5	1.593	1.097–2.314	1.352	0.802–2.281
No history of LVH	83.7	1.767	1.283–2.433	1.483	0.943–2.334
History of LVH	16.3	1.435	0.660–3.120	1.194	0.489–2.919
No diabetes	84.5	1.788	1.285–2.488	1.474	0.945–2.301
Diabetes	15.5	1.173	0.576–2.388	1.105	0.407–2.998
No history of stroke/TIA	81.2	1.534	1.097–2.146	1.313	0.845–2041
history of stroke/TIA	18.8	2.145	1.128–4.081	1.849	0.687–4.972
NO history of LVSD	80.9	1.845	1.320–2.579	1.547	0.944–2.535
history of LVSD	19.1	1.289	0.684–2.432	1.123	0.560–2.252
No history of COPD	88.3	2.025	1.445–2.836	1.556	0.993–2.438
history of COPD	11.7	0.822	0.440–1.536	0.599	0.238–1.509
No Valvular heart disease	83.3	1.720	1.251–2.366	1.435	0.905–2.276
Valvular heart disease	16.7	1.729	0.774–3.862	1.242	0.538–2.866
No history of Bleeding	97.6	1.730	1.284–2.331	1.378	0.922–2.059
History of bleeding	2.4	^a^		^a^	
No ACE inhibitor	73.5	1.806	1.285–2.538	1.396	0.856–2.274
ACE inhibitor	26.5	1.375	0.755–2.504	1.303	0.635–2.676
No β‐blocker	49.6	1.857	1.218–2.830	1.677	0.906–3.104
β‐blocker	50.4	1.583	1.042–2.403	1.236	0.723–2.114
No diuretics	57.5	1.567	1.060–2.315	1.294	0.688–2.433
diuretics	42.5	1.814	1.144–2.877	1.489	0.882–2.515
No digoxin	64.4	2.043	1.388–3.009	1.794	0.981–3.279
Digoxin	35.6	1.228	0.781–1.931	1.101	0.652–1.859

Abbreviations: CAD, coronary artery disease; COPD, chronic obstructive pulmonary disease; HF, heart failure; HTN, hypertension; LVH, left ventricular hypertrophy; LVSD, left ventricular systolic dysfunction; MI, myocardial infarction; TIA, transient ischemic attack.

^a^ Relative risk (95% CI) of history of bleeding was too small to demonstrate.

In the multivariable Cox analysis, advanced age (HR: 3.104, 95% CI: 2.291–4.205), history of heart failure (HR: 1.877, 95% CI: 1.361–2.588), and previous stroke/TIA (HR: 1.388, 95% CI: 1.021–1.888) were the independent predictors of all‐cause mortality in the secondary AF diagnosis group. In contrast, risk factors of all‐cause mortality for the primary AF diagnosis group were advanced age (HR: 3.710, 95% CI: 2.137–6.441), left ventricular systolic dysfunction (HR: 2.754, 95% CI: 1.202–6.306), history of COPD (HR: 3.115, 95% CI: 1.676–5.788), and diabetes (HR: 2.135, 95% CI: 1.092–4.172). For cardiovascular mortality, advanced age (HR: 2.737, 95% CI: 1.802–4.160), a history of heart failure (HR: 3.235, 95% CI: 1.980–5.285) were independent predictors in the secondary AF diagnosis group, while advanced age (HR: 3.460, 95% CI: 1.647–7.267), female gender (HR: 2.597, 95% CI: 1.029–6.558), LVSD (HR: 4.438, 95% CI: 1.658–11.883), history of COPD (HR: 2.482, 95% CI: 1.011–6.092) were risk factors in primary AF diagnosis group (Table 4).

**TABLE 4:** Risk factors of Death for primary or secondary AF diagnosis patients (A: all‐cause mortality, B: cardiovascular mortality)

	Secondary AF diagnosis		Primary AF diagnosis	
	Hazard Ratio	95% CI	Hazard Ratio	95% CI
(A)				
Female gender	0.756	0.563–1.014	1.110	0.628–1.962
Aged ≥ 75	3.104	2.291–4.205	3.710	2.137–6.441
Sustain AF	1.217	0.836–1.771	1.587	0.898–2.805
MI	1.032	0.654–1.628	0.308	0.041–2.323
CAD	0.742	0.542–1.018	1.145	0.651–2.015
HF	1.887	1.361–2.588	0.699	0.342–1.428
HTN	0.935	0.692–1.264	0.953	0.528–1.719
Stroke/TIA	1.388	1.021–1.888	0.950	0.478–1.886
Smoke	0.925	0.650–1.316	0.823	0.393–1.726
LVSD	1.062	0.752–1.501	2.754	1.202–6.306
COPD	1.226	0.873–1.722	3.115	1.676–5.788
Diabetes	1.224	0.869–1.723	2.135	1.092–4.172
Valvular disease	0.976	0.658–1.447	1.528	0.648–3.605
Major bleeding	0.633	0.277–1.445	1.343	0.182–9.907
OAC prescription	0.870	0.594–1.274	0.570	0.239–1.359
(B)				
Female gender	0.892	0.587–1.357	2.597	1.029–6.558
Aged ≥ 75	2.737	1.802–4.160	3.460	1.647–7.267
Sustain AF	1.035	0.608–1.760	2.241	0.944–5.320
MI	1.742	0.949–3.198	^a^	
CAD	0.481	0.298–0.775	1.128	0.522–2.438
HF	3.235	1.980–5.285	0.733	0.303–1.773
HTN	1.234	0.805–1.891	0.825	0.372–1.829
Stroke/TIA	1.151	0.728–1.819	1.000	0.388–2.575
Smoke	1.051	0.649–1.700	1.713	0.609–4.824
LVSD	1.347	0.875–2.074	4.438	1.658–11.883
COPD	1.115	0.677–1.838	2.482	1.011–6.092
Diabetes	1.230	0.755–2.002	2.317	0.912–5.887
Valvular disease	1.174	0.713–1.933	2.081	0.783–5.526
Major bleeding	0.417	0.101–1.720	^a^	
OAC prescription	0.961	0.591–1.562	0.862	0.315–2.359

Note

The acronym was the same as Table 3.

Abbreviation: OAC, oral anticoagulation.

## 4 DISCUSSION

This analysis from the Chinese AF registry shows that ED patient with a secondary diagnosis of AF had remarkably high mortality rate compared with those with a primary diagnosis of AF. The all‐cause mortality risk was increased by 72% in secondary AF diagnosis patients compared with primary AF diagnosis patients after adjustment for the confounders.

To the best of our knowledge, this is the first large outcome study to explore the impact of primary diagnosis on mortality in AF patients. In a pilot study, Atzema et al.(Atzema, Lam, Young, & Kester‐Greene, 2013) described the characteristics and outcomes in a small group of AF patients and found that the crude mortality was three times higher in secondary AF diagnosis patients than in those with a primary diagnosis of AF. Nevertheless, due to the limitation of this single‐center, retrospective study with a portion of incomplete data, the authors emphasized that the conclusions might be inconclusive and further study was warranted. Here we performed a well‐designed, multicenter, prospective work to demonstrate a more convincible result as expected and to further explore the potential risks of mortality in each group.

Patient with a secondary diagnosis of AF was much older and had a worse condition with more concomitant disease compared with primary AF diagnosis patients. This was consistent with the former study (Andersson et al., 2013; Atzema et al., 2013). As we all know, AF is particularly common in elderly people, and any condition that predisposes to left atrial enlargement will associate a rising incidence of AF (Schoonderwoerd, Smit, Pen, & Van Gelder, 2008). Apparently, a number of classical factors, such as heart failure, hypertension, valvular disease, diabetes mellitus, cardiomyopathy, obesity, or thyroid disease, are powerful stimulus for the initiation and development of AF, and this is quite familiar in the clinical practice. In our study, beta‐blockers were less often used in patients with a secondary diagnosis of AF, which may relate to the higher age and more comorbidities. Treatment with digoxin was more frequent among secondary AF diagnosis group and that may reflect the lower heart rates on admission. Due to its narrow therapeutic index and a potential to contribute to life‐threatening arrhythmia, the use of digoxin for rate control in AF patients remains controversial (Hallberg et al., 2007). Especially, two recent post hoc analysis of the AFFIRM data got opposed conclusions on digoxin use and all‐cause mortality (Gheorghiade et al., 2013; Whitbeck et al., 2013). In the present study, digoxin was not associated with mortality neither in primary AF diagnosis group nor in secondary AF diagnosis group in multivariable Cox analysis, and we expect further study to investigate the role of digoxin in the contemporary management of AF patients. There was no significant difference between these two groups on antithrombotic therapy. However, it was worth noting that the oral anticoagulants prescription in our population was much lower than reported from previous study (Nieuwlaat et al., 2005). Under‐treatment with anticoagulation agents is a great challenge, especially in secondary AF diagnosis group which was at high risk of thrombosis.

After adjustment for confounders, the all‐cause mortality risk for patients with a secondary diagnosis of AF remained significantly higher than those with primary AF diagnosis, indicating that secondary AF diagnosis was an independent risk of mortality. The observed difference between patients with AF as a primary diagnosis and as a secondary diagnosis indicated the great influence of concomitant diseases on mortality risk. In our analysis, the top one reason for admission in secondary AF diagnosis patients and the major cause of death for the total study population was heart failure. Atrial fibrillation and heart failure are two of the most prevalent cardiovascular disease conditions. They often coexist and lead to significant morbidity and mortality. Many patients with advanced heart failure develop AF as the severity of heart failure increases. The SOLVD trial (Dries et al., 1998) suggested that the presence of AF tends to worsen the prognosis of patients with asymptomatic and symptomatic left ventricular systolic dysfunction. Analysis from the CHARM program demonstrated that AF is associated with an increased risk of cardiovascular outcomes in patients with heart failure, both in reduced and preserved left ventricular ejection fraction (Olsson et al., 2006). Moreover, incident heart failure has an adverse impact on prognosis in AF independently of other cardiovascular diagnoses and risk factor. AF, particularly when the heart rate is poorly controlled, can lead to the development of dilated cardiomyopathy and heart failure (Suzuki et al., 2012). Clearly, atrial fibrillation is a complex condition and frequently associated with admissions for hypertension, stroke, heart failure, acute coronary syndrome, or infection. Physicians could not ignore the interaction about AF and its concomitant disease. Therefore, we emphasize the importance of focusing on patients as an entirety rather than a single disease entity.

We also analyzed risk factors of mortality in the study population. After adjustment for comorbidities, the independent predictors for all‐cause mortality were advanced age, heart failure, and previous stroke in secondary AF diagnosis group, whereas for primary AF diagnosis patients, advanced age, diabetes, history of left ventricular systolic dysfunction, and chronic obstructive pulmonary disease portended a worse prognosis. Similar conclusions have been drawn on the independent predictors for both groups on cardiovascular mortality. Based on these findings, we propose that clinician should distinguish the primary diagnosis of patients with AF presenting to ED and consider a more powerful therapy in those with above risks. What's more, it is obvious that cardiac function has a strong association with all‐cause mortality in AF patients regardless of the primary diagnosis (Badheka et al., 2011). Thus, we recommended that the echocardiogram was necessary for ED visiting patients with AF.

## 5 LIMITATIONS

We used a large administrative database from the Chinese AF registry for analysis, of which 2.7% were patients with atrial flutter. Typical atrial flutter has a well‐defined macro‐reentrant circuit in the right atrium as its major mechanism and therefore can be relatively easily cured by ablation. Nevertheless, AF and atrial flutter usually coexist and patients with atrial flutter develop AF even subsequently to successful ablation (Perez et al., 2009). Moreover, response to therapy and management approaches for atrial flutter in improvement of survival and reduction of cardiovascular complication is similar to those of AF. So they can be treated as one entity in trials designed to investigate the outcomes. In addition, the anticoagulation rate in the present study was much lower than reported from previous literature (Casciano, Singer, Kwong, Fox, & Martin, 2012). Due to the nature of an observational study that management decisions were made by individual physicians, the snapshot of anticoagulation could just reflect the current status and we underline that an appropriate management of anticoagulation therapy in AF patients was warranted.

## 6 CONCLUSIONS

Patients with secondary diagnosis of AF were associated with an increased 1‐year mortality compared with those with primary AF diagnosis. Physicians should distinguish these two groups and pay attention to their risk factors on treatment.

## CONFLICT OF INTEREST

None.

## AUTHOR CONTRIBUTIONS

Dr Shao contributed to statistical analyses, data interpretation, and drafting and revisions of the manuscript. Dr Yang: contributed to study design and hypothesis, data interpretation, and drafting of the manuscript. Dr Zhu, Dr Yu, and Dr Liu: contributed to drafting and revision of the manuscript.

## ETHICAL APPROVAL

All procedures performed in this study were in accordance with the ethical standards of the institutional and national research committee and with the 1964 Helsinki declaration and its later amendments.
